# Application of Machine Learning in Diagnosis of COVID-19 Through X-Ray and CT Images: A Scoping Review

**DOI:** 10.3389/fcvm.2021.638011

**Published:** 2021-03-25

**Authors:** Hossein Mohammad-Rahimi, Mohadeseh Nadimi, Azadeh Ghalyanchi-Langeroudi, Mohammad Taheri, Soudeh Ghafouri-Fard

**Affiliations:** ^1^Dental Research Center, Research Institute of Dental Sciences, Shahid Beheshti University of Medical Sciences, Tehran, Iran; ^2^Department of Medical Physics and Biomedical Engineering, Tehran University of Medical Sciences (TUMS), Tehran, Iran; ^3^Research Center for Biomedical Technologies and Robotics (RCBTR), Tehran, Iran; ^4^Urology and Nephrology Research Center, Shahid Beheshti University of Medical Sciences, Tehran, Iran; ^5^Department of Medical Genetics, Shahid Beheshti University of Medical Sciences, Tehran, Iran

**Keywords:** COVID-19, machine learning, detection, biomarker, X-ray image

## Abstract

Coronavirus disease, first detected in late 2019 (COVID-19), has spread fast throughout the world, leading to high mortality. This condition can be diagnosed using RT-PCR technique on nasopharyngeal and throat swabs with sensitivity values ranging from 30 to 70%. However, chest CT scans and X-ray images have been reported to have sensitivity values of 98 and 69%, respectively. The application of machine learning methods on CT and X-ray images has facilitated the accurate diagnosis of COVID-19. In this study, we reviewed studies which used machine and deep learning methods on chest X-ray images and CT scans for COVID-19 diagnosis and compared their performance. The accuracy of these methods ranged from 76% to more than 99%, indicating the applicability of machine and deep learning methods in the clinical diagnosis of COVID-19.

## Introduction

First identified in Wuhan, China, severe pneumonia caused by Severe Acute Respiratory Syndrome Coronavirus 2 (SARS-CoV-2) quickly spread all over the world. The resultant disorder was named coronavirus disease (COVID-19) (1, 2). COVID-19 has various clinical symptoms, including fever, cough, dyspnea, fatigue, myalgia, headache, and gastrointestinal complications (3–5). Diagnosis of COVID-19 infection through RT-PCR on nasopharyngeal and throat swab samples has been reported to yield positive results in 30–70% of cases (6, 7). On the other hand, chest CT scans and X-ray images have been reported to have sensitivity values of 98 and 69%, respectively (7–9). The most typical radiological signs in these patients include multifocal and bilateral ground-glass opacities and consolidations, particularly in the peripheral and basal sites (10). However, interpretation of the results of these imaging techniques by expert radiologists might encounter some problems leading to reduced sensitivity (11). Artificial intelligence has recently gained the attention of both clinicians and researchers for the appropriate management of the COVID-19 pandemic (12). As an accurate method, artificial intelligence is able to identify abnormal patterns of CT and X-ray images. Using this method, it is possible to assess certain segment regions and take precise structures in chest CT images facilitating diagnostic purposes. Artificial intelligence methods have been shown to detect COVID-19 and distinguish this condition from other pulmonary disorders and community-acquired pneumonia (13). Both deep learning and machine learning approaches have been used to predict different aspects of the COVID-19 outbreak. Support vector and random forest are among the most applied machine learning methods, while Convolutional Neural Network (CNN), Long Short-Term Memory (LSTM), Generative Adversarial Networks (GAN), and Residual Neural network are among the deep learning methods used in this regard (14). In this study, we reviewed studies which used machine and deep learning methods on chest X-ray images and CT scans for the purpose of COVID-19 diagnosis and compared their performance.

## Methods

### Search Strategy

The research question was: “What are the applications of machine learning techniques and their performances in COVID-19 diagnosis using X-ray images?”. The search of the present review was based on the PICO elements, which were as follows:

- **P (Problem/Patient/Population):** Patients' CT scans and Chest X-rays.
- **I (Intervention/Indicator):** Machine/deep learning models for diagnosis of Covid-19 patients
- **C (Comparison):** Ground truth or reference standards
- **O (Outcome):** Performance measurements including accuracy, AUC score, sensitivity, and specificity.

In other words, we were looking for publications that evaluated the performance of any machine learning or deep learning approaches based on inclusion and exclusion criteria. Studies that used other types of medical image modalities (e.g., ultrasound images) were excluded. An electronic search was conducted on PubMed, Google Scholar, Scopus, Embase, arXiv, and medRxiv for finding the relevant literature. Duplicate studies were removed. Studies that were cited within the retrieved papers were reviewed for finding missing studies. For identifying proper journal papers and conference proceedings, investigators screened the title and abstracts based on inclusion and exclusion criteria independently. Finally, considering the inclusion and exclusion criteria, investigators identified the eligible publications in this stage independently.

### Inclusion Criteria

The following inclusion criteria were used in the selection of the articles: (1) Studies that applied machine learning or deep learning algorithms, (2) Studies that evaluated the measurement of model outcomes in comparison with ground truth or gold standards, and (3) Studies that used algorithms to analyze radiographic images (CT scan, Chest X-ray, etc.).

### Exclusion Criteria

The following studies were excluded: (1) Studies that used any machine learning or deep learning approaches for problems not directly related to the COVID-19 imaging, (2) Studies that used other artificial intelligence techniques or classic computer vision approaches, (3) Studies that did not provide a clear explanation of the machine learning or deep learning model that was used to solve their problem, and (4) Review studies. The latter were excluded as we did not aim to review the data on an original level without any second-hand interpretations (summation, inferences, etc.).

**Figure 1:**
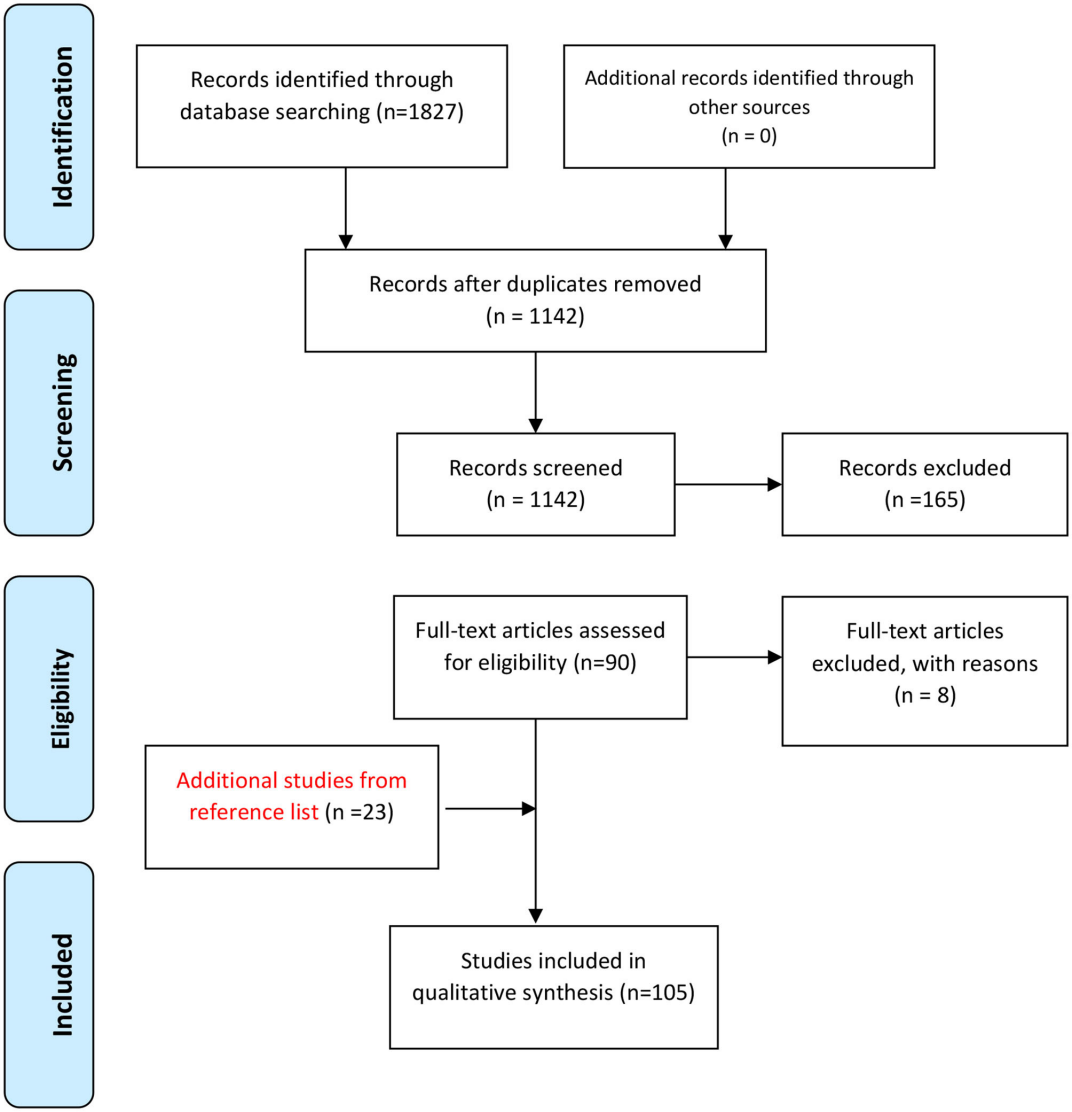
PRISMA (Preferred Reporting Items for Systematic Reviews and Meta-Analyses) chart showing the process of systematic identification, screening, and selection of articles.

Figure 1 shows the flowchart of the study design.

## Results

We obtained 105 studies that used machine or deep learning methods to assess chest images of COVID-19 patients. These studies have used different analytical methods. For instance, Ardakani et al. (15) have assessed radiological features of CT images obtained from patients with COVID-19 and non-COVID-19 pneumonia. They used decision tree, K-nearest neighbor, naïve Bayes, support vector machine, and ensemble classifiers to find the computer-aided diagnosis system with the best performance in distinguishing COVID-19 patients from non-COVID-19 pneumonia. They reported that site and distribution of pulmonary involvement, the quantity of the pulmonary lesions, ground-glass opacity, and crazy-paving as the most important characteristics for differentiation of these two sets of patients. Their computer-aided diagnosis method yielded the accuracy of 91.94%, using an ensemble (COVIDiag) classifier. Alazab et al. (16) have developed an artificial-intelligence method based on a deep CNN to evaluate chest X-ray images and detection of COVID-19 patients. Their method yielded an F-measure ranging from 95 to 99%. Notably, three predicting strategies could forecast the numbers of COVID-19 confirmations, recoveries, and mortalities over the upcoming week. The average accuracy of the prediction models were 94.80 and 88.43% in two different countries. Albahli has applied deep learning-based models on CT images of COVID-19 patients. He has demonstrated a high performance of a Deep Neural Network model in detecting COVID-19 patients and has offered an efficient assessment of chest-related disorders according to age and sex. His proposed model has yielded 89% accuracy in terms of GAN-based synthetic data (17). Automatic detection of COVID-19 based on X-ray images has been executed through the application of three deep learning models, including Inception ResNetV2, InceptionNetV3, and NASNetLarge. The best results have been obtained from InceptionNetV3, which yielded the accuracy levels of 98.63 and 99.02% with and without application of data augmentation in model training, respectively (18). Alsharman et al. (19) have used the CNN method to detect COVID-19 based on chest CT images in the early stages of disease course. Authors have reported high accuracy of GoogleNet CNN architecture for diagnosis of COVID-19. Altan et al. (20) have used a hybrid model comprising two-dimensional curvelet transformation, chaotic salp swarm algorithm, and deep learning methods for distinguishing COVID-19 from other pneumonia cases. Application of their proposed model on chest X-ray images has led to accurate diagnosis of COVID-19 patients (Accuracy = 99.69%, Sensitivity = 99.44% and Specificity = 99.81%). Apostolopoulos et al. (21) have used a certain CNN strategy, namely MobileNet on X-Ray images of COVID-19 patients. This method has yielded more than 99% accuracy in the diagnosis of COVID-19. In another study, Ardakani et al. (22) used 10 CNN strategies, namely AlexNet, VGG-16, VGG-19, SqueezeNet, GoogleNet, MobileNet-V2, ResNet-18, ResNet-50, ResNet-101, and Xception, to differentiate COVID-19 cases from non-COVID-19 patients. They have demonstrated the best diagnostic values for ResNet-101 and Xception, both of them having area under curve (AUC) values higher than 0.99 which is superior to the performance of the radiologist. Das et al. (23) have used the CNN model Truncated InceptionNet to diagnose COVID-19 from other non-COVID and/or healthy cases based on chest X-ray. Their suggested model yielded AUC of 1.0 in distinguishing COVID-19 patients from combined Pneumonia and healthy subjects. Tables 1, 2 summarize the features of studies which adopted machine learning methods in CT images and chest X-ray of COVID-19 patients, respectively.

**Table 1:** Characteristics of papers that used CT images or a combination of X-ray and CT images.

**Author, year**	**Data source**	**Data structure and size**	**Data preprocessing**	**Best model structure(s)**	**Performance measurements (on the best model)**				**References**
					**Accuracy**	**AUC score**	**Sensitivity**	**Specificity**	
Abbasian et al. (2020)	Iran University of Medical Sciences (IUMS)	306 COVID-19 patients; 306 COVID-19 pneumonia (CT images)	Extracting 20 features of CT images	Ensemble	91.94%	0.965	93.54%	90.32%	(15)
Alsharman et al. (2020)	“COVID-CT-dataset”	CT images	Binarization (the separation of the object and background is known as Binarization); Converting input image from 2D Grayscale to 3D Color	GoogleNet CNN	82.14%				(19)
Ardakani et al. (2020)	Private dataset	108 COVID-19 patients; 86 viral pneumonia diseases (CT images)	Converted to the gray-scale Cropped and resized to 60 * 60 pixels	ResNet-101 Xception	Resnet: 99.51% Xception: 99.02% (compared to 86.7% in human)	Resnet: 0.994 Xception: 0.994% (compared to 0.873 in human)	Resnet: 100% Xception: 98.04% (compared to 89.21% in human)	Resnet: 99.02% Xception: 100% (compared to 83.33% in human)	(22)
Aswathy et al. (2020)	“National Cancer Institute and the Cancer Image Archive”	1,763 normal patients; 63 pneumonia patients	Thresholding; Texture-based feature extractionwith a wrapper	CNN	99%	–	–	–	(24)
Bai et al. (2020)	Private dataset	521 COVID-19 patients; 665 other pulmonary diseases (CT images)	Lung segmentation; Generate an 8-bit image for each axial slice by applying Lung windowing to the Hounsfield units	EfficientNet B4	96% (compared to 85% in human)	0.95	95% (compared to 79% in human)	96% (compared to 88% in human)	(11)
Bridge et al. (2020)	“Toy dataset;” “Italian Society of Radiology;” “Shenzhen Hospital X-Ray dataset;” “ChestX-Ray8;” “COVID-CT-Dataset”	129 COVID-19 patients; 62,267 normal patients; 5,689 pneumonia patients (X-ray images) 30 COVID-19 patients; 1,919 normal patients (CT images)	Using the GEV activation function for unbalanced data	Inception V3	100%	–	100%	100%	(25)
Butt et al. (2020)	Not mentioned	219 images from 110 COVID-19 patients; 399 normal patients (CT images)	Image processing method base on HU values	3D CNN	–	0.996	98.2%	92.2%	(26)
Dey et al. (2020)	“COVID-19 CT segmentation dataset;” “Chest X-rays (Radiopaedia)”	200 COVID-19 patients; 200 normal patients (grayscale lung CTI images)	Segmenting lung area related to pneumonia infection; Extracting CWT, DWT, EWT features from original image and Haralick, Hu moments from binary segmented area Feature selection based on statistical tests	KNN	87.75%	–	89.00%	86.50%	(27)
El Asnaoui et al. (2020)	COVID-19 X-ray image database developed by Cohen JP; Kermany et al. (28)	2,780 Bacterial pneumonia patients; 1,493 Coronavirus patients; 231 COVID-19 patients; 1,583 normal patients (X-ray and CT images)	Intensity Normalization; Contrast Limited Adaptive Histogram Equalization	Inception ResNetV2; Densnet201	Inception-ResNetV2: 92.18% Densnet201: 88.09%	Inception-ResNetV2: 0.920 Densnet201: 0.879	Inception-ResNetV2: 92.11% Densnet201: 87.99%	Inception-ResNetV2: 96.6% Densnet201: 94.00%	(29)
Han et al. (2020)	“COVID-19 hospitals in Shandong Province”	79 COVID-19 patients; 100 pneumonia patients; 130 normal patients (CT images)	Data augmentation	AD3D-MIL	97.9%	0.99	97.9%	97.9%	(30)
Harmon et al. (2020)	Private dataset	386 COVID-19 patients; 1,011 negative COVID-19 patients (CT images)	Lung segmentation; clipping images to HU range (−1,000, 500); Data augmentation (flipping, rotation, image intensity and contrast adjustment, adding random Gaussian noise);	Hybrid 3D based on Densnet-121	90.8%	–	84%	93%	(31)
Hasan et al. (2020)	“Radiopaedia and the cancer imaging archive websites”	118 COVID-19 patients; 96 pneumonia patients; 107 normal patients (CT images)	Histogram Thresholding; Dilation; Hole Filling	LSTM	99.68%	–	100%	–	(32)
Hu et al. (2020)	“Hospital of Wuhan Red Cross Society;” “Shenzhen Hospital;” “TCIA dataset;” “Cancer Centre Archive (TCIA) Public Access;” “MD Anderson Cancer Centre;” “Memorial Sloan-Kettering Cancer Center;” “MAASTRO clinic”	150 COVID-19 patients; 150 pneumonia patients; 150 normal patients (CT images)	Data augmentation	CNN	96.2%	0.970	94.5%	95.3%	(33)
Jaiswal et al. (2020)	“The SARS-CoV-2 CT scan dataset”	1,262 COVID-19 patients; 1,230 non-COVID-19 patients (CT images)	Data augmentation (rotation up to 15, slant-angle of 0.2, horizontal flipping, filling new pixels as “nearest” for better robustness)	DenseNet201	96.25%	0.97	96.29%	96.21%	(34)
Kang et al. (2020)	“Tongji Hospital of Huazhong University of Science and Technology;” “China-Japan Union Hospital of Jilin University;” “Ruijin Hospital ofShanghai Jiao Tong University”	1,495 COVID-19 patients; 1,027 community-acquired pneumonia (CAP) patients (CT images)	Normalization; Standardization	NN	93.90%	–	94.60%	91.70%	(35)
Lessmann et al. (2020)	“Emergency wards of an Academic center and teaching hospital in the Netherlands in March and April 2020”	237 COVID-19 patients; 606 normal patients (CT images)	Resampling; Normalization	CORADS-AI	–	0.95	85.7%	89.8%	(36)
Li et al. (2020)	Private	1,296 COVID-19 patients; 1,325—patients; 1,735 community-acquired (CT images)	Segmenting lung area with U-net	COVNet (ResNet-50)	–	0.96	90%	96%	(13)
Li et al. (2020)	More than 10 medical centers between Nov. 11th, 2010 and Feb. 9th, 2020	305 images from 251 COVID-19 patients; 872 images from 869 pneumonia patients; 1,498 images from 1,475 non-pneumonia patients (CT images)	DL-based algorithm Image processing method base on HU values; Data augmentation	3D ResNet-18	Recall = 88% Precision = 89.6% F1 score = 87.8%				(37)
Liu et al. (2020)	Private	73 COVID-19 patients; 27 general pneumonia patients (CT images)	ROI delineation based on ground-glass opacities (GGOs); 13 gray level co-occurrence matrix (GLCM) features, 15 gray level-gradient co-occurrence matrix (GLGCM) features, and six histogram features were extracted; Feature selection by ReliefF;	An ensemble of bagged tree (EBT)	94.16%	0.99	88.62%	100%	(38)
Mei et al. (2020)	Private	419 COVID-19 patients 486 non-COVID-19 patients (CT images)	Selecting pertinent slices by image segmentation to detect parenchymal tissue; Segmenting lung in CT images;	ResNet-18	79.6%	0.86	83.6%	75.9%	(39)
Panwar et al. (2020)	“COVID-chest X-ray;” “SARS-COV-2 CT-scan;” “Chest X-Ray Images (Pneumonia);”	206 COVID-19 patients; 364 Pneumonia patients (X-ray and CT images)	–	VGG-19	95.61% (COVID-19 vs. Pneumonia)	–	96.55% (COVID-19 vs. Pneumonia)	95.29% (COVID-19 vs. Pneumonia)	(40)
Pathak et al. (2020)	2 different COVID-19 datasets of chest-CT images	CT images	–	Deep bidirectional long short-term memory network with mixture density network (DBM)	96.19% (multi-class)	0.96 (multi-class)	96.22% (multi-class)	96.16% (multi-class)	(41)
Pathak et al. (2020)	“COVID-19 open datasets of chest CT images”	413 COVID-19 patients; 439 normal or pneumonia infected patients (CT images)	–	ResNet-50	93.01%	–	91.45%	94.77%	(41)
Peng et al. (2020)	Collected from PMC	606 COVID-19 patients; 222 Influenza; 397 Normal or other disease patients (CT images)	–	DenseNet121	–	0.87	72.3%	85.2%	(42)
Pu et al. (2020)	Private	498 COVID-19 patients; 497 community-acquired pneumonia (CAP) (CT images)	Data augmentation [rotation, translation, vertical/horizontal flips, Hounsfield Unit (HU) shift, smoothing (blurring) operation, Gaussian noise]	3D CNNs	99%	0.7	–	–	(43)
Raajan et al. (2020)	X-ray images on public medical Github repositories; Kaggle chest X-ray database	349 images from 216 COVID-19 patients; 1,341 Normal patients (CT images)	Normalization	ResNet-16	95.09%	–	100%	81.89%	(44)
Rajaraman et al. (2020)	“Pediatric CXR dataset;” “RSNA CXR dataset;” “Twitter COVID-19 CXR dataset;” “Montreal COVID-19 CXR dataset”	313 COVID-19 patients; 7,595 pneumonia of unknown type patients; 2,780 bacterial pneumonia; 7,595 Normal patients (X-ray images)	Median filtering; Normalization; Standardization	Inception-V3	99.01%	0.997	98.4%	–	(45)
Sakagianni et al. (2020)	COVID-19 articles on medRxiv and bioRxiv	349 COVID-19 patients; 397 non-COVID-19 patients (CT images)	–	AutoML Cloud Vision	–	0.94	88.31%	–	(46)
Sharma (2020)	Dataset from Italian Society of Medical and Interventional Radiology; COVID-CT available in GitHub; Dataset from hospitals in Moscow, Russia; Dataset from SAL Hospital, Ahmedabad, India;	800 COVID-19 patients; 600 Viral Pneumonia; 800 normal patients (CT images)	Ground-glass opacities (GGO), consolidation and pleural effusion are the features	ResNet	91%	–	92.1%	90.29%	(47)
Singh et al. (2020)	Not mentioned	CT images	–	Multi-objective differential evolution (MODE) based CNN	90.22%	–	91.17%	89.23%	(48)
Song et al. (2020)	Private (two hospitals in China);	98 COVID-19 patients; 103 non-COVID-19 pneumonia (CT images)	–	BigBiGAN	–	0.972	92%	91%	(49)
Wang et al. (2020)	Private	1,315 COVID-19 patients; 2,406 ILD patients; 936 Normal patients (CT images)	Lobe Segmentation by 3D-Unet; Converting CT numbers to grayscale	PA-66 model	93.3%	0.973	97.6%	–	(50)
Wang et al. (2020)	COVID-19 dataset (private); CT-epidermal growth factor receptor (CT-EGFR) dataset (private);	754 COVID-19 patients; 271 bacterial pneumonia 29 viral pneumonia; 42 Other pneumonia (CT images) *The CT-EGFR dataset was used for auxiliary training of the DL system	Lung segmentation; Using a fully automatic DL model (DenseNet121-FPN); suppress the intensities of non-lung areas inside the lung ROI;	COVID-19Net (DenseNet-like architecture)	Test-set1: 78.32% Test-set2: 80.12%	Test-set1: 0.87 Test-set2: 0.88	Test-set1: 80.39% Test-set2: 79.35%	Test-set1: 76.61% Test-set2: 81.16%	(51)
Warman et al. (2020)	“Public sources”	606 COVID-19 patients; 224 viral pneumonias patients; 74 Normal patients (CT images)	Data augmentation	YOLOv3 model	96.80%	0.966	98.33%	94.95%	(52)
Wu et al. (2020)	Private	368 COVID-19 patients; 127 other pneumonia (CT images)	Lung region in each axial, coronal and sagittal CT slices were segmented using threshold segmentation and morphological optimization algorithms; The slice with the most pixels in the segmented lung area from each of the axial, coronal and sagittal views was selected as the inputs of the deep learning network;	Multi-view fusion ResNet50 architecture	76%	0.819	81.1%	61.5%	(53)
Xu et al. (2020)	Private “Hospitals in Zhejiang Province, China.”	219 images from 110 COVID-19 patients; 224 Influenza-A viral pneumonia patients; 175 Normal patients (CT images)	Image processing method base on HU values	3D CNN segmentation model	86.7%	–	86.7%	–	(54)
Xu et al. (2020)	Private	432 COVID-19 patients; 76 other viral pneumonia; 350 bacterial pneumonia; 418 normal patients (CT images)	Sampling 5 subsets of CT slices from all sequential images of one CT case to picture the infected lung regions.	3D-Densenet	–	0.98	97.5% (differentiating COVID-19 from three types of non-COVID-19 cases) (compared to 79% in human)	89.4% (differentiating COVID-19 from three types of non-COVID-19 cases) (compared to 90% in human)	(55)
Yan et al. (2020)	Private	416 images from 206 COVID-19 patients; 412 common pneumonia patients (CT images)	Transferring image slices to JPG; Normalization	MSCNN	97.7%	0.962	99.5%	95.6%	(56)
Yang et al. (2020)	Private	146 COVID-19 patients; 149 normal patients (CT images)	For patients, images containing round-glasses opacity (GGO), GGO with consolidation was selected; for healthy control, every 3 slices containing pulmonary parenchyma were selected; Lung windowing is performed over all image slices;	DenseNet	92% (compared to 95% in human)	0.98	97% (compared to 94% in human)	87% (compared to 96% in human)	(57)
Yu et al. (2020)	Private	202 COVID-19 patients (CT images)	–	DenseNet-201 with the cubic SVM model	95.2%	0.99	91.87%	96.87%	(58)
Al-Karawi et al. (2020)	“COVID-CT-Dataset”	275 COVID-19 patients; 195 normal patients (CT images)	Adaptive winner filter followed by inversion; Feature extraction by the FFT-spectrum	SVM	95.37%	–	95.99%	94.76%	(59)
Alom et al. (2020)	Publicly available datasets; “Kaggle repository”	3,875 pneumonia patients; 1,341 normal patients (X-Ray images) 178 COVID-19 patients; 247 normal patients (CT images)	Data augmentation; Adaptive Thresholding Approach	IRRCNN model; NABLA-3 network model	X-ray images: 84.67% CT images: 98.78%	0.93	–	–	(60)
Barstugan et al. (2020)	From the Italian Society of Medical and Interventional Radiology	150 COVID-19 patients (CT images)	13 features were extracted by Gray Level Size Zone Matrix (GLSZM)	SVM	98.77%	–	97.72%	99.67%	(61)
Chen et al. (2020)	Private dataset	25,989 images from 51 COVID-19 patients; 20,107 images from 55 normal patients (retrospective dataset); 13,911 images from 27 consecutive patients (prospective dataset) (CT images)	Filtering	Deep learning model	Retrospective dataset: 95.24%; Prospective dataset: 92.59% (per patient)	–	Retrospective dataset: 100%; Prospective dataset: 100% (per patient)	Retrospective dataset:93.55%; Prospective dataset: 81.82% (per patient)	(62)
Farid et al. (2020)	Kaggle database	51 COVID-19 patients (CT images)	Feature extraction (MPEG7 Histogram Filter, Gabor Image Filter, Pyramid of Rotation-Invariant Local Binary Pattern, Fuzzy 64-bin Histogram Image Filter); Feature selection by composite hybrid feature selection	CHFS-Stacked (jrip, RF) with Naïve Bayes classifier	96.07%	–	–	–	(63)
Gozes et al. (2020)	Dataset1:ChainZ; Dataset2: Private; Dataset3: ChainZ;	50 suspicious COVID-19 patients from dataset1 used for training; 56 COVID-19 patients; 51 normal patients (CT images) used for testing	Data augmentation (rotation, horizontal flips and cropping)	Resnet-50-2D	–	0.996	98.2%	92.2%	(64)
Jin et al. (2020)	Three centers in China; “LIDC-IDRI;” “Tianchi-Alibaba;” “CC-CCII”	2,529 images from 1,502 COVID-19 patients; 1,338 images from 1,334 CAP patients; 135 images from 83 influenza-A/B patients; 258 images from 258 normal patients (CT images)	–	CNN	–	0.977	90.19%	95.76%	(65)
Jin et al. (2020)	Data from three different centers in Wuhan; Data from three publicly available databases, LIDC-IDRI26, Tianchi-Alibaba27, and CC-CCII18;	1,502 COVID-19 patients; 83 influenza-A/B patients; 1,334 CAP patients except for influenza; 258 healthy subjects (CT images)	Segmenting lung area with U-net	ResNet152	–	0.971	90.19%	95.76%	(66)
Hosseinzadeh Kassani et al. (2020)	COVID-19 X-ray image database developed by Cohen JP; “Kaggle chest X-ray database;” “Kaggle RSNA Pneumonia Detection dataset”	117 COVID-19 patients; 117 normal patients (X-Ray images); 20 COVID-19 patients; 20 normal patients (CT images)	Normalization	DenseNet121 with Bagging tree classifier	99%	–	96%	–	(67)
Ozkaya et al. (2020)	From the Italian Society of Medical and Interventional Radiology	53 COVID-19 patients (CT images)	Feature vectors obtained from Pre-trained VGG-16, GoogleNet and ResNet-50 networks and fusion method; Feature ranking by *t*-test method	SVM	98.27%	–	98.93%	97.60%	(68)
Shi et al. (2020)	From Tongji Hospital, Shanghai Public Health Clinical Center, and China-Japan Union Hospital (all in China)	183 COVID-19 patients; 5,521 Pneumonia patients (CT images)	Segmentation by a deep learning network (VB-Net)	Infection size-aware random forest	87.9%	0.942	90.7%	83.3%	(69)
Song et al. (2020)	From the Renmin Hospital of Wuhan University	88 COVID-19 patients (CT images)	We extracted the main regions of lungs and filled the blank of lung segmentation with the lung itself	Details Relation Extraction neural network	86%	0.96	96%	–	(3)
Wang et al. (2020)	Private dataset	44 COVID-19 patients; 55 Pneumonia patients (CT images)	Random selection of ROI; Feature extraction using Transfer Learning	Fully connected network and combination of Decision tree and Adaboost	82.9%	0.90	81%	84%	(6)
Zheng et al. (2020)	Private dataset	313 COVID-19 patients; 229 non-COVID-19 patients (CT images)	Data augmentation; Producing lung masks by a trained UNet	3D deep convolutional neural network	90.8%	0.959	–	–	(70)

*Data Source: The source(s) that images were acquired from, Data Structure and Size: Number of images, image modalities, sample groups, Data Preprocessing: cleaning, Instance selection, normalization, transformation, feature extraction, selection, etc. The product of data preprocessing is the final training set, Best Model Structure(s): Best machine algorithm or deep learning model reported in the selected paper based on its performance, Performance Measurements (on the best model): The measurement of the model's output performance based on accuracy, sensitivity, specificity, and AUC score*.

**Table 2:** Characteristics of papers that used X-ray images.

**Author, year**	**Data source**	**Data structure and size**	**Data preprocessing**	**Best model structure(s)**	**Performance measurements (on the best model)**				**References**
					**Accuracy**	**AUC score**	**Sensitivity**	**Specificity**	
Alazab et al. (2020)	Kaggle database	70 COVID-19 patients 28 normal patients (X-ray images)	Augmented to 1,000 images	VGG-16	F1 Score: 0.99				(16)
Albahli et al. (2020)	“ChestX-ray8” combined with the few samples of rare classes from the Kaggle challenge	108,948 X-ray images of 32,717 unique patients. Including 15 kinds of chest disease	Data augmentation (rotation, height shift, zoom, horizontal flip)	ResNet	89%	–	–	–	(17)
Albahli et al. (2020)	Open source COVIDx dataset	850 COVID-19 patients; 500 non-COVID-19 pneumonia cases; 915 normal patients (X-ray images)	Data augmentation	InceptionNetV3	99.02%	–	–	–	(18)
Altan et al. (2020)	Not mentioned	7,980 chest X-ray image (2,905 real raw 5,075 synthetic chests X-ray images)	Data augmentation; The feature matrix is formed by 2D Curvelet transformation Coefficients; Optimizing the coefficients in the feature matrix with the CSSA	Hybrid model	99.69%	–	99.44%	99.81%	(20)
Apostolopoulos et al. (2020)	COVID-19 X-ray image database developed by Cohen JP; Common Bacterial and Viral Pneumonia X-ray Images by Kermany et al.; Public datasets (Radiological Society of North America, Radiopaedia, and the Italian Society of Medical and Interventional Radiology); “NIH Chest X-ray Dataset”	455 COVID-19 patients; 910 viral pneumonia; 2,540 other pulmonary diseases (X-ray images)	Data augmentation (randomly rotated by a maximum of 10° and randomly shifted horizontally or vertically by a maximum of 20 pixels toward any direction)	MobileNet v2	99.18%	–	97.36%	99.42%	(21)
Apostolopoulos et al. (2020)	X-ray images on public medical Github repositories; “Radiological Society of North America;” “Radiopaedia, and Italian Society of Medicine and Interventional Radiology”	Dataset 1: 224 COVID-19 patients; 700 bacterial pneumonia patients; 504 normal patients (X-ray images) Dataset 2: 224 Covid-19 patients; 714 bacterial and viral pneumonia patients; 504 normal patients (X-ray images)	-	MobileNet v2	96.78%	–	98.66%	96.46%	(71)
Brunese et al. (2020)	COVID-19 image data collection; COVID-19 X-ray image database developed by Cohen JP; “ChestX-ray8;” “NIH Chest X-ray Dataset”	250 COVID-19 patients; 2,753 other pulmonary diseases; 3,520 normal patients (X-Ray images)	Data augmentation (15 degrees rotation clockwise or counterclockwise)	VGG-16	96% (comparison between COVID-19 and other pulmonary diseases)	–	87% 96%	94% 98%	(72)
Chowdhury et al. (2020)	Kaggle chest X-ray database; “Italian Society of Medical and Interventional Radiology COVID-19 database;” “Novel Corona Virus 2019 Dataset;” GitHub database; “COVID-19 Chest imaging at thread reader;” “RSNA-Pneumonia-Detection-Challenge”	423 COVID-19 patients; 1,485 viral pneumonia patients; 1,579 normal patients (X-ray images)	Data augmentation	CNN	99.7%	–	99.7%	99.55%	(73)
Civit-Masot et al. (2020)	COVID-19 and Pneumonia Scans Dataset	132 COVID-19 patients; 132 normal patients; 132 Pneumonia patients (X-ray images)	Histogram equalization	VGG16	85%	–	85%	92%	(74)
Das et al. (2020)	COVID-19 collection; “Kaggle CXR collection;” “Tuberculosis collections;” “U.S. National Library of Medicine;” “National Institutes of Health;” Pneumonia collections	162 COVID-19 patients; 1,583 normal patients	Histogram matching	Truncated Inception Net	100% (Pneumonia collections)	1.0	100%	100%	(23)
Elaziz et al. (2020)	COVID-19 X-ray image database developed by Cohen JP; “Chest X-Ray Images Pneumonia;” Italian Society of Medical and Interventional Radiology COVID-19 DATABASE;	219 COVID-19 patients; 1,341 negative COVID-19 patients (X-ray images)	Feature extraction by Fractional Multichannel Exponent Moments (FrMEMs); Feature selection by modified Manta-Ray Foraging Optimization based on differential evolution	KNN	98.09	–	98.91	–	(75)
Hassantabar et al. (2020)	“COVID-CT-Dataset”	315 COVID-19 patients; 367 non-COVID-19 patients (X-ray images)	–	CNN	93.2%	–	96.1%	99.71%	(76)
Islam et al. (2020)	“GitHub;” “Radiopaedia;” “Cancer Imaging Archive;” “Italian Society of Radiology;” “Kaggle repository;” NIH dataset	1,525 COVID-19 patients; 1,525 pneumonia patients; 1,525 normal patients (X-ray images)	Normalization	CNN-LSTM	99.4%	0.999	99.3%	99.2%	(77)
Khan et al. (2020)	“Covid-chestxray-dataset” “Chest X-Ray Images (Pneumonia)”	284 COVID-19 patients; 330 Pneumonia Bacterial 327 Pneumonia Viral; 310 normal patients (X-ray images)	Random under-sampling (to overcome the unbalanced data problem)	CoroNet (based on Xception)	89.6%	–	89.92%	96.4%	(78)
Khuzani et al. (2020)	“GitHub”	140 COVID-19 patients; 140 non-COVID-19 pneumonia patients; 140 normal patients (X-ray images)	PCA method; Min-Max Normalization; Adaptive Histogram Equalization	ML	94%	0.91	100%	–	(79)
Ko et al. (2020)	Private; Italian Society of Medical and Interventional Radiology COVID-19 DATABASE;	1,194 COVID-19 patients; 1,442 non-pneumonia patients; 1,357 Pneumonia patients (X-ray images)	Data augmentation (rotation, zoom)	FCONet (ResNet-50)	99.58%	–	99.58%	100%	(80)
Loey et al. (2020)	COVID-19 X-ray image database developed by Cohen JP	69 COVID-19 patients; 79 pneumonia bacterial patients; 79	Data augmentation	Googlenet	80.56% (Four classes)	–	80.56%	–	(81)
Mahmud et al. (2020)	Private	1,583 normal patients; 1,493 non-COVID viral pneumonia; 2,780 bacterial pneumonia; 305 COVID-19 patients (X-ray images)	–	CovXNet (CNN based architecture)	90.2% (multi-class)	0.911 (multi-class)	89.9% (multi-class)	89.1% (multi-class)	(82)
Martínez et al. (2020)	COVID-19 X-ray image database developed by Cohen JP	120 COVID-19 patients; 120 normal patients (X-ray images)	Data augmentation; Normalization	NASNet-type convolutional	97%	–	97%	97%	(83)
Minaee et al. (2020)	COVID-19 X-ray image database developed by Cohen JP; “ChexPert dataset”	40 COVID-19 patients; 3,000 normal patients (X-ray images)	Regularization	SqueezeNet	97%	–	97.5%	97.8%	(84)
Narayan Das et al. (2020)	COVID-19 X-ray image database developed by Cohen JP; “ChestX-ray8”	125 COVID-19 patients; 500 pneumonia patients; 500 normal patients (X-ray images)	–	Xception	97.4%	0.986	97.09%	97.29%	(85)
Nour et al. (2020)	“Public COVID-19 radiology database;” “Italian Society of Medical and Interventional Radiology;” “COVID-19 Database;” “Novel Corona Virus 2019 Dataset;” “COVID-19 positive chest X-ray images from different articles;”	219 COVID-19 patients; 1,345 Viral Pneumonia patients; 1,341 Normal patients (X-ray images)	Data augmentation	CNN	97.14%	0.995	94.61%	98.29%	(86)
Novitasari et al. (2020)	GitHub and Kaggle	102 COVID-19 patients; 204 Pneumonia and Normal patients (X-ray images)	Feature extraction by Googlenet, Resnet18, Resnet50, Resnet101; Feature selection by PCA, Relief;	SVM	97.33% (multi class)	–	96%	98%	(87)
Oh et al. (2020)	“Japanese Society of Radiological Technology;” “SCR database;” “U.S. National Library of Medicine”	180 COVID-19 patients; 20 Viral Pneumonia patients; 54 pneumonia bacterial patients; 57 Tuberculosis patients; 191 Normal patients (X-ray images)	Data normalization; Data type casting; Histogram equalization; Gamma correction	(FC)-DenseNet103	88.9%	–	85.9%	96.4%	(88)
Ozturk et al. (2020)	COVID-19 X-ray image database developed by Cohen JP; “ChestX-ray8;”	(X-ray images)		DarkCovidNet inspired by the DarkNet architecture	87.02%	–	85.35%	92.18%	(89)
Pandit et al. (2020)	COVID-19 X-ray image database developed by Cohen JP; Kaggle chest X-ray database	224 COVID-19 patients; 700 pneumonia bacterial patients; 504 Normal patients (X-ray images)	Data augmentation	VGG-16	92.53% (Three class output)	–	86.7%	95.1%	(90)
Panwar et al. (2020)	COVID-19 X-ray image database developed by Cohen JP; Radiopedia.org website; Kaggle chest X-ray database	142 COVID-19 patients; 142 other (“Normal” “Bacterial Pneumonia” and “Viral Pneumonia”) (X-ray images)	Data augmentation	nCOVnet	88.10%	0.880	97.62%	78.57%	(40)
Pereira et al. (2020)	“RYDLS-20;” Radiopedia Encyclopedia “Chest X-ray14”	90 COVID-19 patients; 1,000 Normal patients; 10 MERS patients; 11 SARS patients; 10 Varicella patients; 12 Streptococcus patients; 11 Pneumocystis patients (X-ray images)	Resampling algorithms; Fusion techniques;	Pre-trained CNN	F1 score = 89%				(91)
Rahaman et al. (2020)	COVID-19 X-ray image database developed by Cohen JP; “Chest X-Ray Images (pneumonia)”	260 COVID-19 patients; 300 Pneumonia; 300 Normal patients (X-ray images)	Data augmentation (rotate, shift, shear, zoom, horizontal and vertical flip)	VGG19	89.3%	–	89%	–	(92)
Rahimzadeh et al. (2020)	“Covid chestxray dataset;” “RSNA pneumonia detection challenge”	180 COVID-19 patients; 6,054 Pneumocystis patients; 8,851 Normal patients (X-ray images)	Data augmentation	Xception ResNet50V2 concatenated	91.4%	–	80.53%	99.56%	(93)
Rajaraman et al. (2020)	Pediatric CXR dataset; RSNA CXR dataset; CheXpert CXR dataset; NIH CXR-14 dataset; Twitter COVID-19 CXR dataset; Montreal COVID-19 CXR dataset;	4,683 Bacterial Pneumonia; 3,883 Viral Pneumonia (X-Ray images)	Segmenting lung area with dilated dropout U-Net; Image thresholding to remove very bright pixels; In-painting missing pixels using the surrounding pixel values; Using median-filter to remove noise and preserve edges;	VGG-16	94.05%	0.96	98.77%	86.24%	(45)
Rajaraman et al. (2020)	“Pediatric CXR dataset;” “RSNA CXR dataset;” “Twitter COVID-19 CXR dataset;” “Montreal COVID-19 CXR dataset”	313 COVID-19 patients; 7,595 pneumonia of unknown type patients; 2,780 bacterial pneumonia; 7,595 Normal patients (X-ray images)	Median Filtering; Normalization; Standardization	Inception-V3	99.01%	0.997	98.4%	–	(45)
Sethy et al. (2020)	X-ray images on public medical Github repositories; Kaggle chest X-ray database	127 COVID-19 patients; 127 Pneumonia patients; 127 Normal patients (X-ray images)	–	ResNet50 plus SVM	98.66%	–	95.33%	–	(94)
Shibly et al. (2020)	COVID-19 X-ray image database developed by Cohen JP; “RSNA pneumonia detection challenge dataset;” Kaggle chest X-ray database; “COVIDx”	183 COVID-19 patients; 5,551 Pneumonia patients; 8,066 Normal patients (X-ray images)	–	Faster R-CNN	97.36%	–	97.65%	–	(95)
Togaçar et al. (2020)	COVID-19 X-ray image database developed by Cohen JP; Kaggle COVID-19 dataset created by a team of researchers from Qatar University, medical doctors from Bangladesh, and collaborators from Pakistan and Malaysia.	295 COVID-19 patients; 98 Pneumonia; 65 normal patients (X-ray images)	Restructuring images using the Fuzzy Color technique and stacking them with the original images; Feature extracting using deep learning models (MobileNetV2, SqueezeNet) using the Social Mimic optimization method;	SVM	100%	–	100%	100%	(96)
Toraman et al. (2020)	COVID-19 X-ray image database developed by Cohen JP	231 COVID-19 patients; 1,050 Pneumonia patients; 1,050 Normal patients (X-ray images)	Data augmentation;	Convolutional capsnet	97.24% (Binary class)	–	97.42%	97.04%	(97)
Tsiknakis et al. (2020)	COVID-19 X-ray image database developed by Cohen JP; Dataset originated from the QUIBIM imagingcovid19 platform database and various public repositories, including RSNA, IEEE, RadioGyan and the British Society of Thoracic Imaging; Publicly available X-ray dataset of patients with pneumonia;	137 COVID-19 patients; 150 Virus Pneumonia; 150 Bacteria Pneumonia; 150 normal patients (X-ray images)	Data augmentation (rotation, shear, zoom)	Inception V3	76% (multi-class)	0.93 (multi-class)	93% (multi-class)	91.8% (multi-class)	(98)
Tuncer et al. (2020)	GitHub website; Kaggle chest X-ray database	87 COVID-19 patients; 234 Normal patients (X-ray images)	Converting X-ray image to grayscale; ResExLBP and IRF based method	SVM	100%	–	98.29%	100%	(99)
Ucar et al. (2020)	“COVID chest X-ray dataset;” “Kaggle chest X-ray pneumonia dataset;”	403 COVID-19 patients; 721 normal patients (X-ray images)	Data augmentation (noise, shear, brightness increase, brightness decrease)	Bayes-SqueezeNet	98.26% (multi-class)	–	–	99.13% (multi-class)	(100)
Vaid et al. (2020)	Set of lately published articles; NIH dataset	181 COVID-19 patients; 364 Normal patients (X-ray images)	Normalization	VGG-19	96.3%	–	97.1%	–	(101)
Waheed et al. (2020)	“IEEE Covid Chest X-ray dataset;” “COVID-19 Radiography Database” “COVID-19 Chest X-ray Dataset;”	403 COVID-19 patients; 721 normal patients (X-ray images)	Data augmentation using CovidGAN	VGG16	95%	–	90%	97%	(102)
Yildirim et al. (2020)	“COVID-19 Chest X-Ray dataset;” Kaggle chest X-ray database	136 COVID-19 patients; 162 Pneumonia patients; 245 Normal patients (X-ray images)	–	Hybrid model	96.30%	–	96.30%	98.73%	(103)
Yoo et al. (2020)	“COVID-Chest XrayDataset;” Eastern Asian Hospital; Shenzen data;	162 COVID-19 Patients; 162 TB patients; 162 Non-TB patients (X-ray images)	Data augmentation (rotated, translated, and horizontally flipped)	ResNet18	95% Average of (COVID-19/TB) and (COVID-19/non-TB)	0.95 Average of (COVID-19/TB) and (COVID-19/non-TB)	97% Average of (COVID-19/TB) and (COVID-19/non-TB)	93% Average of (COVID-19/TB) and (COVID-19/non-TB)	(104)
Ghoshal et al. (2020)	COVID-19 X-ray image database developed by Cohen JP; “Kaggle chest X-ray database”	68 COVID-19 patients; 2,786 Bacterial Pneumonia patients; 1,504 Viral Pneumonia patients; 1,583 normal patients (X-Ray images)	Standardization; Data augmentation	Bayesian ResNet50V2 model	89.82%	–	–	–	(105)
Hall et al. (2020)	“X-ray images on public medical Github repositories;” “Radiopaedia;” “Italian Society of Medical and Interventional Radiology (SIRM)”	135 COVID-19 patients; 320 Viral and Bacterial Pneumonia patients (X-Ray images)	Data augmentation	Resnet50 and VGG16 plus CNN	91.24%	0.94	–	–	(106)
Hammoudi et al. (2020)	“Chest XRay Images (Pneumonia) dataset;” COVID-19 X-ray image database developed by Cohen JP;	148 Bacterial pneumonia; 148 Viral pneumonia; 148 Normal patients (X-Ray Images)	–	DenseNet169	95.72%	–	–	–	(107)
El-Din Hemdan et al. (2020)	COVID-19 X-ray image database developed by Cohen JP; COVID-19 X-ray image database by Dr. Adrian Rosebrock	25 COVID-19 patients; 25 normal patients (X-Ray images)	Scaling to 224*224 pixels; One-hot encoding	COVIDX-Net (VGG19 and DenseNet201 models)	VGG19 = 90%; DenseNet201 = 90%	VGG19 = 0.90; DenseNet201 = 0.90	VGG19 = 100%; DenseNet201 = 100%	–	(108)
Jain et al. (2020)	“Chest XRay Images (Pneumonia) dataset;” COVID-19 X-ray image database developed by Cohen JP;	250 COVID-19 patients; 300 Bacterial pneumonia; 350 Viral pneumonia; 315 Normal patients (X-Ray Images)	Normalize images according to the images in the ImageNet database; Data augmentation (rotation and Gaussian blur);	ResNet50	97.77%	–	97.14%	–	(109)
Luz et al. (2020)	“COVIDx dataset;” “RSNA Pneumonia Detection Challenge dataset;” “COVID-19 image data collection”	183 COVID-19 patients; 5,521 Pneumonia patients; 8,066 normal patients (X-Ray images)	Intensity normalization; Data augmentation	EfficientNet B3	93.9%	–	96.8%	–	(110)
Ozkaya et al. (2020)	From the Italian Society of Medical and Interventional Radiology	53 COVID-19 patients (CT images)	Feature vectors obtained from Pre-trained VGG-16, GoogleNet and ResNet-50 networks and fusion method; Feature ranking by *t*-test method	SVM	98.27%	–	98.93%	97.60%	(68)
Ozturk et al. (2020)	“covid-chestxray-dataset available at: https://github.com/ieee8023/covid-chestxray-dataset”	4 ARds images, 101 COVID images, 2 No finding images, 2 pneumocystis-pneumonia images, 11 Sars images, and 6 streptococcus (X-Ray images)	Data augmentation; SMOTE oversampling; creating feature vectors with sAE and PCA; feature extraction by feature vectors, Gray Level Co-occurrence Matrix, Local Binary Gray Level Co-occurrence Matrix, Gray Level Run Length Matrix, and Segmentation-based Fractal Texture Analysis	SVM	94.23%	0.99	91.88%	98.54%	(111)
Wang et al. (2020)	COVIDx dataset	266 COVID-19 patients; 5,536 Pneumonia patients; 8,066 normal patients (X-Ray images)	–	COVID-Net Network Architecture using a “lightweight residual projection-expansion- projection-extension design pattern” (Customized CNN)	93.3%		91.0%	–	(1)
Zhang et al. (2020)	X-COVID, OpenCOVID	599 COVID-19 patients; 2,107 non-COVID-19 patients (non-viral pneumonia and healthy) (X-Ray images)	Data augmentation; Feature extraction using EfficientNet	Confidence-aware anomaly detection	78.57%	0.844	77.13%	78.97%	(112)

*Data Source: The source(s) that images were acquired from, Data Structure and Size: Number of images, image modalities, sample groups, Data Preprocessing: cleaning, Instance selection, normalization, transformation, feature extraction, selection, etc. The product of data preprocessing is the final training set, Best Model Structure(s): Best machine algorithm or deep learning model reported in the selected paper based on its performance, Performance Measurements (on the best model): The measurement of the model's output performance based on accuracy, sensitivity, specificity, and AUC score*.

## Discussion

Machine and deep learning methods have been proven as valuable strategies to assess massive high-dimensional characteristics of medical images. CT or X-Ray findings of COVID-19 patients have similarities with other atypical and viral pneumonia diseases. Therefore, machine and deep learning methods might facilitate automatic discrimination of COVID-19 from other pneumonia conditions. The differential diagnosis of COVID also includes drug-induced diseases or immune pneumonitis. However, most of the studies reviewed here lack these kinds of samples. This point is the limitation of these studies. Different methods, such as Ensemble, VGG-16, ResNet, InceptionNetV3, MobileNet v2, Xception, CNN, VGG16, Truncated Inception Net, and KNN, have been used for the purpose of assessment of chest images of COVID-19 patients. Notably, the application of these methods on X-rays has offered promising results. Such a finding is particularly important since X-rays are easily accessible and low cost. These methods not only can diagnose COVID-19 patients from non-COVID pneumonia cases, but can also predict the severity of COVID-19 pneumonia and the risk of short-term mortality. In spite of the low expense of X-ray compared with CT images, the numbers of studies that assessed these two types of imaging using machine/deep learning methods are not meaningfully different. However, few studies have used these methods on both types of imaging (25, 29, 40). CNN-based methods have achieved accuracy values above 99% in classifying COVID-19 patients from other cases of pneumonia or related disorders, as reported by several independent studies, suggesting these strategies as screening methods for initial evaluation of COVID-19 cases.

Although both deep learning and machine learning strategies can be used for the mentioned purpose, they differ in some respects. For instance, deep learning methods usually need a large amount of labeled training data to make a concise conclusion. However, machine learning can apply a small amount of data delivered by users. Moreover, deep learning methods need high-performance hardware. Machine learning, on the other hand, needs features to be precisely branded by users, deep learning generates novel features by itself, thus requires more time to train. Machine learning classifies tasks into small fragments and subsequently combines obtained results into one conclusion, whereas deep learning resolves the problems using end-to-end principles.

Several studies have diagnosed COVID-19 patients through the application of machine learning methods rather than using deep learning methods by retrieving the features from the images. These studies have yielded high recognition outcomes and have the advantage of high learning speed (12). Pre-processing is an essential step for reducing the impacts of intensity variations in CT slices and getting rid of noise. Subsequent thresholding and morphological operations have also enhanced the analytical performance. Data augmentation and histogram equalization are among the most applied preprocessing methods.

One of the most promising approaches used in the included studies was transfer learning. Transfer learning is defined as using model knowledge on a huge dataset (which is referred to as the “pre-trained model”) and transferring it to use on a new problem. This is very useful in settings like medical imaging, where there is a limited number of labeled data (113). Previous studies showed favorable outcomes of the transfer learning approaches in medical imaging tasks (114, 115). Among the included studies, Bridge et al. (25) even reached 100% classification accuracy on COVID-19 using the pre-trained InceptionV3.

The availability of public databases of CT and X-ray images of patients with COVID-19 has facilitated the application of machine learning methods on large quantities of clinical images and execution of training and verification steps. However, since these images have come from various institutes using different scanners, preprocessing of the obtained data is necessary to make them uniform and facilitate further analysis (12). Appraisal of demographic and clinical data of COVID-19 patients and their association with CT/ X-ray images features as well as the accuracy of machine learning prediction methods would provide more valuable information in the stratification of COVID-19 patients. Moreover, one of the major challenges of deep learning models in medical applications is its unexplainable features due to its black-box nature, which should be solved (116). Future studies can focus on approaches that provide interpretation besides black-box predictions.

## Conclusion

Deep and machine learning methods have high accuracy in the differentiation of COVID-19 from non-COVID-19 pneumonia based on chest images. These techniques have facilitated the automatic evaluation of these images. However, deep learning methods suffer from the absence of transparency and interpretability, as it is not possible to identify the exact imaging feature that has been applied to define the output (13). As no single strategy has the capacity to distinguish all pulmonary disorders based merely on the imaging presentation on chest CT scans, the application of multidisciplinary approaches is suggested for overcoming diagnostic problems (13).

## Data Availability Statement

The original contributions presented in the study are included in the article/supplementary material, further inquiries can be directed to the corresponding authors.

## Author Contributions

HM-R, MN, and AG-L collected the data and designed the tables. MT and SG-F designed the study, wrote the draft, and revised it. All the authors read the draft and approved the submitted version.

## Conflict of Interest

The authors declare that the research was conducted in the absence of any commercial or financial relationships that could be construed as a potential conflict of interest.
